# Multicentre clinical radiotherapy audit in rectal cancer: results of the IROCA project

**DOI:** 10.1186/s13014-020-01648-7

**Published:** 2020-08-27

**Authors:** Magdalena Fundowicz, Artur Aguiar, Carla Lopes de Castro, Maria Glòria Torras, Letizia Deantonio, Ewelina Konstanty, Marta Kruszyna-Mochalska, Miquel Macia, Eugeni Canals, Monica Caro, Carla Pisani, Dorota Zwierzchowska, Jaume Molero, Arantxa Eraso, Joana Lencart, Carles Muñoz-Montplet, Luisa Carvalho, Marco Krengli, Julian Malicki, Ferran Guedea

**Affiliations:** 1 Greater Poland Cancer Centre, Garbary 15 St, 61-866 Poznan, Poland; 2 Instituto Português de Oncologia; do Porto FG, EPE (IPO-Porto), Rua Dr. António Bernardino de Almeida, 4200-072 Porto, Portugal; 3 Institut Català d’Oncologia, L’Hospitalet, Avinguda Granvia de l’Hospitalet, 199-203, 08908 L’Hospitalet de Llobregat, Barcelona, Spain; 4 “Amedeo Avogadro” - Rettorato, Università degli Studi del Piemonte Orientale (UNIUPO), via Duomo, 6 - 13100 Vercelli, Novara, Italy; 5 Deparment of Electroradiology, University of Medical Sciences, Fredry 10, 61-701 Poznan, Poland; 6 Institut Català d’Oncologia, Avinguda de França, S/N 17007, Girona, Spain; 7 Institut Català d’Oncologia, Ctra. Canyet s/n 08916, Badalona, Spain

## Abstract

**Purpose:**

To perform a clinical audit to assess adherence to standard clinical practice for the diagnosis, treatment, and follow-up of patients undergoing radiotherapy for rectal cancer treatment in four European countries.

**Materials and methods:**

Multi-institutional, retrospective cohort study of 221 patients treated for rectal cancer in 2015 at six European cancer centres. Clinical indicators applicable to general radiotherapy processes were evaluated. All data were obtained from electronic medical records.

**Results:**

The audits were performed in the year 2017. We found substantial inter-centre variability in adherence to standard clinical practices: 1) presentation of cases at departmental clinical sessions (range, 0–100%) or multidisciplinary tumour board (50–95%); 2) pretreatment MRI (61.5–100%) and thoracoabdominal CT (15.0–100%). Large inter-centre differences were observed in the mean interval between biopsy and first visit to the radiotherapy department (range, 21.6–58.6 days) and between the first visit and start of treatment (15.1–38.8 days). Treatment interruptions ≥ 1 day occurred in 43.9% (2.5–90%) of cases overall. Treatment compensation was performed in 2.1% of cases. Treatment was completed in the prescribed time in 55.7% of cases.

**Conclusions:**

This multi-institutional clinical audit revealed that most centres adhered to standard clinical practices for most of the radiotherapy processes-related variables assessed. However, the audit revealed marked inter-centre variability for certain quality indicators, particularly inconsistent record keeping. Multiple targets for improvement and/or harmonisation were identified, confirming the value of routine clinical audits to detect potential deviations from standard clinical practice.

## Introduction

Rectal cancer accounts for approximately one third of all cancers diagnosed in Europe [1]. In most cases, treatment consists of a combination of radiotherapy, chemotherapy, and surgery [2]. The use of high-dose ionising radiation, particularly in the pelvic region, requires strict quality control measures to achieve optimal outcomes while minimizing toxicity to surrounding healthy organs [3]. In recent years, the role of clinical audits in improving quality has been increasing recognized. However, to date only a limited number have been conducted in the field of radiation oncology [4–8], and even fewer have specifically focused on rectal cancer [9–12].

In this context, six European comprehensive cancer centres joined together in an international project known as IROCA (*Improving Quality in Radiation Oncology through Clinical Audits;* *https://iroca.eu*) to carry out clinical audits of the radiotherapy process [10]. This project was, in part, inspired by previous experience at two of these hospitals, which performed a clinical audit to identify best practices and thereby improve and optimize radiotherapy delivery at those institutions [9].

The main aim of the present study was to assess adherence to standard clinical practice, defined by expert consensus based on clinical guidelines and protocols, for key radiotherapy and organizational processes in the treatment of rectal cancer at six large cancer centres in Europe using a multicentre clinical audit. Here we report the results of that audit, which specifically assessed adherence to clinical protocols and international guidelines, including the diagnosis, treatment, and follow-up of patients.

## Material and methods

This was a multi-institutional, retrospective cohort study involving a representative sample of patients (40 patients/centre) diagnosed with rectal cancer at six participating cancer centres: the Wielkopolskie Centrum Onkologii (WCO) in Poznan, Poland; the Instituto Português de Oncologia (IPO) in Porto, Portugal; the Università degli Studi del Piemonte Orientale (UNIUPO) in Novara, Italy; and the three hospitals that form the Institut Català d’Oncologia (ICO) in Spain (located in Barcelona, Badalona, and Girona).

### Study design

For the present study, quality indicators and clinical indicators were selected by a working group of radiation oncologists and medical physicists from the participating centres, led by a senior clinician in rectal cancer at each institution after a review of the relevant guidelines, as described below. This same team also developed the clinical audit model, which was broadly based on models used in two previous clinical audits conducted by members of the IROCA group [9, 11].

### Selection of quality indicators and clinical parameters

First, the working group reviewed the relevant literature [5, 13], including the main clinical guidelines for staging and treatment of rectal cancer, as well as institutional and national guidelines in place at each participating centre. Next, based on the review og guidelines and on the group’s previous experience in performing clinical audits, we selected a set of clinical indicators applicable to the general approach and treatment of rectal cancer.

Inclusion criteria were: 1) confirmed diagnosis of rectal cancer (RE: CIM-9:154.1), 2) no metastatic or recurrent disease, and 3) treatment with preoperative intent radiotherapy. To minimize the risk of bias, all patients who met the inclusion criteria were assigned an identification number and then randomly selected for inclusion. However, because two of the centres (UNIUPO and IPO) had fewer than 40 patients who met all inclusion criteria, randomization was not performed in those centres.

All patients were treated in accordance with the clinical guidelines in place at the time of treatment (year 2015) [13]. Short-course radiotherapy (SCRT, 25Gy in 5 fractions) was indicated for patients ≥70 years of age, with tumours located in the middle third of the rectum, or those without involvement of the mesorectal fascia with surgery within 1 week of completion of therapy. Long-course radiotherapy (LCRT) was indicated for all other cases: radiotherapy - 50.4 Gy in 28 fractions with chemotherapy (5-fluorouracil) in the first and the fourth week of treatment.

### Selection of quality indicators

The indicators selected were designed to assess the appropriateness of the tests performed and the quality of the diagnostic reports, particularly the distance from the tumour to the mesorectal fascia (rCRM), based on high-resolution magnetic resonance imaging (MRI). Other indicators were as follows: presentation of cases to a multidisciplinary tumour board (MTB), suitability of and adherence to the prescribed treatment (radiotherapy dose, fraction, and duration), number of imaging verifications performed, postoperative circumferential resection margin (ypCRM), appropriateness of clinical follow-up, and presence and registration of adverse effects. The complete list of quality indicators is shown in Table 1.

**Table 1:** Rectal radiotherapy indicators

**Diagnostic phase**	
% patients diagnosed at different hospital	
% patients with pre-treatment MRI, pelvic ultrasound, thoraco–abdominal CT	
% patients with TNM, MRI staging	
Time between biopsy and first visit at RO	
Time between first visit at RO department and beginning of radiotherapy	
% patients evaluated pre-treatment in RO department clinical session	
% patients presented to Multidisciplinary Tumour Board (MTB)	
**Treatment phase**	
% patients included in clinical trial	
% patients Long Course Radiotherapy (LCRT)	
% patients Short Course Radiotherapy (SCRT)	
% patients with 3DCRT, VMAT, IMRT	
% patients with simultaneous-integrated boost (SIB)	
% patients immobilized	
% patients completing treatment in the prescribed time	
% patients with treatment interruption of EBRT, due to patient-related reasons, due to centre-related reasons, with dose compensation	
Overall treatment time (time between first and last day of treatment)	
% patients presented to the second MTB, to the MTB after surgery	
% patients with restaging MRI, US pelvic, TNM	
**Pathology report**	
% patients with post-surgery ypT, ypN staging	
% patients with adjuvant chemotherapy	
**Side effects**	
**Acute (< 6 months) and chronic (≥ 6 months) adverse effects ≥ grade 3**	
% patients with rectitis, cystitis-urethritis, neutropenia, diarrhea	
**Patient follow up**	
Regular follow-up (≥ 2 visits per annum) after the treatment	
% patients with relapse, mortality	

### Clinical audit

The audits were performed during a 3-month period (April–June) in the year 2017. These audits were carried out by external evaluators unaffiliated with the participating centres to prevent bias and to maintain consistency in data collection. All data were obtained from patient medical records and entered into a centralized online database. The clinical audit involved a review of the clinical records of 40 patients per centre treated with preoperative radiotherapy +/−chemotherapy for rectal cancer during the year 2015.

### Statistical analysis

Sample size was estimated by assuming a reference proportion of 50% for any variable, with a minimum difference between two hospitals defined as 25%, with an alpha risk of 0.05 and beta of 0.10. The resulting sample size per hospital was 40 cases. The Chi square test for categorical variables was used to compare results among hospitals. The SPSS-IBM statistical software program, v.21 (IBM, Armonk, NY; USA) was used to perform the statistical analysis.

## Results

### Patient characteristics

There were no significant differences between the cohorts at the six participating institutions with regard to patient age, disease stage, diagnosis, or treatment. Similarly, the six institutions all used the same treatment planning system and machines.

A total of 221 patients were included. All centres included 40 patients except two centres (UNIUPO and IPO), which had fewer than 40 patients (22 and 39, respectively). Most patients (*n* = 135; 61.1%) were male and the mean patient age was 65.1 years. There were no significant between-centre differences in the patients in any of the following variables: age, histology, disease stage. Patient distribution by disease stage was as follows: stage I (n = 1, 0.5%), stage IIA (*n* = 16, 7.2%), stage IIIA/B (*n* = 143, 64.7%), and stage IIIC (*n* = 22, 10%). Staging data was missing in 39 cases (17.6%), mainly from a single centre (*n* = 35). The most common disease stage was stage III, accounting for 72.7–80% of patients at each centre.

### Diagnostic phase

As Table 2 shows, most patients (81.4%) were diagnosed at a different hospital. Pretreatment MRI was performed in 87.3% of patients (range, 61.5–100% per centre). Thoracoabdominal computed tomography (CT) was performed in 81.9% of cases. In some centres, all patients were presented at the departmental clinical session; however, inter-centre variability was wide, ranging from 0 to 100% of cases (overall: 63.8%). Similarly, most cases (75.6%) were presented to the MTB (range, 50–95%). The mean interval between biopsy and the initial visit to the radiotherapy department was 37.6 days (range, 21.6–58.6). The mean interval between the first visit and initiation of radiotherapy was 22 days, but varied widely (range, 15.1–38.8).

**Table 2:** Diagnostic phase indicators

INDICATORS	ICO-B	ICO-G	ICO H	IPO	NO	WCO	All		
	N (%)	N (%)	N (%)	N (%)	N (%)	N (%)	N	%	P
**Diagnosed at a different hospital**	23 (57.5)	33 (82.5)	37 (92.5)	38 (97.4)	12 (54.5)	37 (92.5)	180	81.4	0.000
**Pre-treatment MRI**	34 (85.0)	40 (100)	40 (100)	24 (61.5)	18 (81.8)	37 (92.5)	193	87.3	0.000
**Pre-treatment pelvic US**	16 (40.0)	3 (7.5)	17 (42.5)	17 (43.6)	5 (22.7)	3 (7.5)	61	27.6	0.000
**Pre-treatment thoracoabdominal CT**	40 (100)	40 (100)	39 (97.5)	37 (94.9)	19 (86.4)	6 (15.0)	181	81.9	0.000
**TNM staging**	40 (100)	39 (97.5)	40 (100)	35 (89.7)	22 (100)	39 (97.5)	215	97.3	0.046
**MRI staging**	36 (90)	34 (85.0)	36 (90)	4 (10.3)	0 (0.0)	38 (95.0)	148	67.0	0.000
**Presented to MTB**	25 (62.5)	20 (50.0)	34 (85.0)	39 (100)	11 (50.0)	38 (95.0)	167	75.6	0.000
**Presented at pre-treatment RO clinical session**	40 (100)	40 (100)	39 (97.5)	0 (0.0)	22 (100)	0 (0.0)	141	63.8	0.000
**Time between biopsy and first visit at RO (±4) [days]**	32.6	30.5	39.2	58.6	45.9	21.6	210	37.6	< 0.001
**Time between first visit at RO and beginning of RT [days]**	15.1	16.5	19.7	16.0	28.5	38.8	221	22.0	< 0.001

### Treatment phase

Overall, 6.3% of patients (range, 0–25%) were included in a clinical trial. As Table 3 shows, most patients (75.1%) were treated with long-course radiotherapy (LCRT), while 22.6% underwent short-course radiotherapy (SCRT). Most patients (65.2%) were treated with three-dimensional radiotherapy (3D-RT), with the remaining patients (34.9%) undergoing either volumetric arc radiotherapy (VMAT) or intensity-modulated radiotherapy (IMRT). A simultaneous-integrated boost (SIB) was administered in 9.5% of patients. Immobilization was used in nearly all cases (98.2%), with very little variation between centres.

**Table 3:** Treatment phase indicators

INDICATORS	ICO-B	ICO-G	ICO H	IPO	NO	WCO	All		
	N (%)	N (%)	N (%)	N (%)	N (%)	N (%)	N	%	P
**Patients included in clinical trial**	3 (7.5)	0 (0.0)	10 (25.0)	0 (0.0)	1 (4.5)	0 (0.0)	14	6.3	0.000
**Long Course Radiotherapy (LCRT)**	33 (82.5)	40 (100)	32 (80.0)	31 (79.5)	11 (50.0)	24 (60.0)	171	77.4	0.000
**Short Course Radiotherapy (SCRT)**	7 (17.5)	0 (0.0)	8 (20.0)	8 (20.5)	11 (50.0)	16 (40.0)	50	22.6	0.000
**3DCRT**	32 (80.0)	40 (100)	29 (72.5)	4 (10.3)	0 (0.0)	39 (97.5)	144	65.1	0.000
**VMAT**	8 (20.0)	0 (0.0)	11 (27.5)	2 (5.1)	6 (27.3)	1 (2.5)	28	12.7	0.000
**IMRT**	0 (0.0)	0 (0.0)	0 (0.0)	33 (84.6)	16 (72.7)	0 (0.0)	49	22.2	0.000
**SIB**	0 (0.0)	9 (22.5)	10 (25.0)	1 (2.6)	0 (0.0)	1 (2.5)	21	9.5	0.000
**Treatment completed in prescription time**	20 (50.0)	4 (10.0)	10 (25.0)	36 (92.3)	14 (63.6)	39 (97.5)	123	55.7	0.000
**RT treatment interruption**	20 (50.0)	36 (90.0)	29 (72.5)	3 (7.7)	8 (36.4)	1 (2.5)	97	43.9	0.000
**Treatment interruption: patient reason**	4 (20.0)	2 (5.6)	1 (3.4)	0 (0.0)	4 (50.0)	1 (100.0)	12	12.4	0.000
**Treatment interruption: center-related**	17 (85.0)	35 (97.2)	28 (96.6)	3 (100.0)	5 (62.5)	0 (0.0)	88	90.7	0.001
**Treatment compensation for interruptions**	0 (0.0)	1 (2.8)	0 (0.0)	0 (0.0)	0 (0.0)	1 (100.0)	2	2.1	0.000
**Presented to 2nd MTB**	18 (45.0)	20 (50.0)	22 (55.0)	3 (7.7)	1 (4.5)	3 (7.5)	67	30.3	0.000
**Restaging MRI**	10 (25.0)	28 (70.0)	18 (45.0)	0 (0.0)	8 (36.4)	37 (92.5)	101	45.7	0.000
**Restaging TNM**	4 (10.0)	20 (50.0)	9 (22.5)	0 (0.0)	0 (0.0)	29 (72.5)	62	28.1	0.000
**Presented to MTB after surgery**	2 (5.0)	23 (57.5)	30 (75.0)	28 (71.8)	5 (22.7)	39 (97.5)	127	57.5	0.000

Treatment was completed within the prescribed time (i.e., with no unexpected interruptions) in 55.7% of cases Treatment interruptions occurred in 43.9% of cases, most commonly due to centre-related reasons (90.7% of cases). The number of days of interruption ranged from 1 to 5 days. Treatment compensation was performed in 2.1% of cases (including interruptions ≥1 day), as most centres did not compensate for missed treatments.

Following completion of SCRT or LCRT and prior to surgery, restaging MRI and TNM were performed, respectively, in 45.7% (range, 0–92.5%) and 28.1% (0–72.5%) of cases. After induction radiotherapy +/− chemotherapy (prior to surgery), 30.3% (4.5–55%) of patients were presented to a second MTB. Postoperatively, 57.5% of patients were presented to the MTB, with significant inter-centre variation (5–97.5%).

### Follow up

Follow up was considered to be “regular” if patients had at least 2 follow-up visits annually. As our data show, most patients (88.2%) met this criterion (Table 4). The follow-up rate was lower in some countries because it was performed at a different centre and thus not registered on the medical records at the participating centre.

**Table 4:** Patient follow-up indicators

INDICATORS	ICO-B	ICO-G	ICO H	IPO	NO	WCO	All		
	N (%)	N (%)	N (%)	N (%)	N (%)	N (%)	N	%	P
**Regular follow-up after treatment**	40 (100.0)	36 (90.0)	39 (97.5)	38 (97.4)	16 (72.7)	26 (65.0)	195	88.2	0.000
**Relapse**	10 (25.0)	14 (38.9)	8 (20.5)	9 (23.7)	6 (37.5)	1 (3.8)	48	24.6	0.038
**Deceased**	6 (15.0)	5 (12.6)	5 (15.5)	3 (7.7)	2 (9.1)	2 (5.0)	23	10.4	0.718

Table 5 shows the pathological findings and treatment-related adverse effects. Acute and chronic adverse effects were observed in 12.3 and 8.2% of cases, respectively.

**Table 5:** Adverse effects and pathological findings indicators

INDICATORS	ICO-B	ICO-G	ICO H	IPO	NO	WCO	All		
	N (%)	N (%)	N (%)	N (%)	N (%)	N (%)	N	%	P
**Post-surgery ypT**	33 (82.5)	36 (90.0)	40 (100.0)	33 (84.6)	16 (72.7)	38 (95.0)	196	88.7	0.013
**Post-surgery ypN**	34 (85.0)	36 (90.0)	38 (95.0)	33 (84.6)	16 (72.7)	38 (95.0)	195	88.2	0.086
**Patients with adjuvant treatment – chemotherapy**	28 (70.0)	35 (87.5)	27 (67.5)	28 (71.8)	8 (36.4)	20 (50.0)	146	66.1	0.000
**SIDE EFFECTS (≥ grade 3)**									
Acute (< 6 mo)									
**Rectitis**	4 (10.0)	1 (2.5)	0 (0.0)	0 (0.0)	1 (4.5)	2 (5.0)	8	3.6	0.153
**Cystitis-urethritis**	0 (0.0)	0 (0.0)	1 (2.5)	0 (0.0)	0 (0.0)	0 (0.0)	1	0.5	0.474
**Neutropenia**	5 (12.5)	0 (0.0)	0 (0.0)	2 (5.1)	0 (0.0)	0 (0.0)	7	3.2	0.006
**Diarrhea**	0 (0.0)	4 (10.0)	3 (7.5)	1 (2.6)	1 (4.5)	2 (5.0)	11	5.0	0.385
Chronic (> 6 mo)									
**Rectitis**	0 (0.0)	0 (0.0)	0 (0.0)	0 (0.0)	0 (0.0)	0 (0.0)	0	0.0	–
**Cystitis-urethritis**	0 (0.0)	1 (2.5)	2 (5.0)	0 (0.0)	0 (0.0)	0 (0.0)	3	1.4	0.279
**Neutropenia**	1 (2.5)	2 (5.0)	3 (7.5)	1 (2.6)	0 (0.0)	0 (0.0)	7	3.2	0.414
**Diarrhea**	3 (7.5)	0 (0.0)	3 (7.5)	2 (5.1)	0 (0.0)	0 (0.0)	8	3.6	0.184

## Discussion

This multicentre, international clinical audit was performed to assess adherence to generally-accepted standard clinical practice for rectal cancer radiotherapy across six European comprehensive cancer centres. Although most centres adhered to standard practices for most of the variables, we observed substantial inter-centre variability for several of the quality indicators, most notably: 1) presentation of cases at the departmental clinical session (range, 0–100%) and the MTB (50–95%); 2) performance of pretreatment MRI (61.5–100%) and thoracoabdominal CT (15.0–100%); 3) mean interval between biopsy and first visit to the radiotherapy department (21.6–58.6 days) and between the first visit and start of radiotherapy (15.1–38.8 days). Treatment interruptions ≥ 1 day occurred in 43.9% (2.5–90%) of cases overall. Although some of this variability was expected and acceptable, the audit identified multiple areas that could be targeted for improvement and/or harmonisation. The most commonly observed deviation from good practice was a failure to register all relevant clinical events and data on patient medical records. The key findings of the clinical audit are presented in Table 6 and the main inter-institutional differences observed in this clinical audit are discussed below.

**Table 6:** Recommended steps for improvement and/or harmonization for selected indicators/processes

PROCESS OR INDICATOR	FINDING	RECOMMENDED ACTION
*DIAGNOSTIC AND PRE-TREATMENT PHASE*		
Presentation to MTB	● 75.6% of cases presented to MTB (range, 50–95%).	● Present all cases to MTB ● Implement quality control measures to ensure registration of data in 100% of cases
Presentation at departmental clinical session	● 63.8% of cases presented	● Establish clear criteria for presenting cases to clinical sessions ● Consider using mini-tumour boards comprised of radiation oncologists specialising in the specific tumour type/location
Staging MRI	● 67.0% of cases (range, 10.3 to 95%)	● Implement measures to raise MRI-based staging to 100%
Participation in clinical trial	● 6.3% (range, 0 to 25%)	● At centres with low rates, seek to increase the percentage of patients participating in clinical trials
*TREATMENT PHASE*		
Median time between biopsy and first visit to RT	● 37.6 days (range, 21.6–58.6)	● Implement measures to reduce this time interval
Median time elapsed between first visit and start of radiotherapy	● 22 days (range, 15.1–38.8)	● Implement measures to reduce this time interval
Treatment interruptions	● 43.9% of cases (EBRT), range 2.5–90%	● Implement measures to reduce interruptions at centres with a high percentage of treatment interruptions ● Consider working on Saturdays and holidays
Treatment compensation rates	● 2.1% of cases compensated (range, 0 to 100%) ● Data not registered in some cases	● Implement measures to increase compensation rates ● Apply quality control measures to ensure registration of data in 100% of cases
Treatment completed in prescribed time	●55.7% of treatments completed in prescribed time ± 4 days (range, 10–97.5%)	● Apply procedures to raise rate to 100% ● Consider working on Saturdays and holidays
*FOLLOW-UP PHASE*		
Registration of AEs missing	● Data missing in 11.2% of cases	● Implement quality control measures to ensure registration of data in 100% of cases

Abbreviations: MTB, multidisciplinary tumour board; MRI, magnetic resonance imaging; AE, adverse events; RT, radiotherapy

### Preoperative MRI/CT

Clinical guidelines for preoperative MRI and CT at the time these patients were treated [13] recommended that all patients diagnosed with colorectal cancer undergo contrast-enhanced thoracoabdominal CT to estimate disease stage, and MRI to assess the risk of local recurrence. In our study, more than 80% of patients (Table 2) underwent staging MRI (87.3%) and thoracoabdominal CT (81.9%) prior to treatment, indicating adherence to standard clinical practice in most cases. However, some institutions had lower rates for these measures, perhaps partially attributable to a failure to register the findings on the clinical record in some cases. In addition, due to resource limitations and high demand, some of the participating centres reported using ultrasound as an alternative to MRI in periods of peak demand to avoid prolonged waiting times for MRI. While most centres performed thoracoabdominal CT in all (or nearly all) cases, one centre (WCO) only evaluated 15% of cases with this technique (the remaining patients underwent chest x-ray and abdominal ultrasound) due to long waiting times for CT during the study period and due to the limitations of this technique.

The distance from the tumour to the rCRM is predictive of local recurrence, with larger distances associated with lower recurrence rates [14]. However, as our data show, in many cases (47.1%), rCRM was not evaluated. Moreover, in some centres, this was measured in only 2.2% of patients. Similarly, although the distance from the tumour to the anal verge was measured in most (77.8%) cases, one centre only measured this distance in 18% of patients. Ideally, both of these measures—rCRM and distance to anal verge—should be performed in 100% of patients. These suboptimal rates in some centres point to a clear target for improvement.

### Presentation of cases to the MTB and departmental clinical sessions

Overall, more than three-fourths of all patients were presented to the MTB, although this varied widely among centres, with one centre presenting only 50% of patients and two other centres presenting 95%. One likely explanation for these differences is the large proportion (> 80%) of cases diagnosed externally, as these patients would have been presented to the MTB at the diagnosing hospital. Nonetheless, this finding was not unexpected given the lack of a generally-accepted protocol for presenting patients to the MTB and our results probably reflect variability in clinical practice across Europe. Nonetheless, this treatment-related question merits greater discussion and perhaps harmonisation. Part of this inter-centre variability could be due to legitimate inter-centre differences in the organizational models of the MTBs. Although a few studies have questioned whether it is really necessary to present all patients to a MTB [15], we believe—as recent studies have shown [16]—that this is important because it may alter the diagnosis or treatment plan in a substantial proportion (20–50%) of cases. The main concerns about the value of the MTP appear to be the structure and composition of the board itself and the thoroughness of decision-making and follow up. According to ESMO recommendations [2], the MTB should consist of a dedicated multidisciplinary team involving radiologists, surgeons, radiation oncologists, medical oncologists, and pathologists.

We also observed marked inter-centre differences in the presentation of cases at departmental clinical sessions: while four centres presented 100% of cases, two centres did not present any. These findings indicate a stark contrast in internal departmental organization and philosophy or policy among the centres. This issue also deserves greater discussion and, potentially, closer harmonisation. These findings underscore the value of performing a clinical audit such as this to identify and discuss inter-centre differences in clinical practice and protocols that would otherwise be overlooked, despite the potential relevance of these aspects to patient outcomes.

### SCRT vs. LCRT

The optimal neoadjuvant regimen for resectable rectal cancer patients is highly controversial. Surveys have reported regional differences in Europe [17], with the data suggesting a preference for SCRT (25 Gy in 5 fractions) in Scandinavian countries while LCRT appears to be preferred in other parts of Europe and in the United States. Our data indicate the presence of regional differences among the participating centres. For example, SCRT was performed in 50% of patients in the centres located in Italy and Poland versus only 20% in Spain and Portugal. Nevertheless, overall, LCRT was much more common than SCRT in our study, with 75% of patients undergoing LCRT. To date, the totality of the evidence suggests that neither technique provides superior outcomes in terms of local control, survival, late toxicity, or quality of life [18]. However, some centres prefer SCRT because it is easier to administer and reduces the overall treatment time. Moreover, it also reduces the risk of treatment interruption, as we observed in this study and discuss below.

### Waiting times between biopsy / first visit and start of radiotherapy

Waiting times are perhaps among the most important quality indicators, as excessive delays between diagnosis and treatment initiation can negatively impact treatment outcomes by increasing the risk of tumour growth and metastasis [19]. We found high variability in the time interval between biopsy and the first visit to the radiotherapy department, which ranged from 22 to 59 days (mean, 37.6). Similar variability was observed with respect to the interval between the first visit and the start of radiotherapy, a finding that indicates substantially longer waiting times in some centres. Numerous factors can account for these inter-centre differences, but it seems probable that the most important cause is an imbalance between available resources (e.g., linear accelerators) and demand.

Most patients included in this study were diagnosed at a different hospital, which explains why the wait time for biopsy results and the time to the first visit at the radiation oncology department were longer in those patients compared to patients diagnosed at the same cancer centre. Other factors that may play a role in increasing wait times include the need to perform additional tests, especially if staging is performed by other specialists (e.g., surgeons, clinical oncologists, gynaecologists), inefficient processes within the health care system, limited resources, and treatment-related factors such as comorbidities and auxiliary therapy [20, 21]. Studies have shown that longer waiting times are often—although not always—associated with worse outcomes [22] and can also increase psychological stress on the patient [23]. Although no standard waiting times have yet been established, national regulations in some individual countries stipulate the maximum time interval. For example, in Poland, the maximum wait (established in 2017) is 14 days from treatment selection to initiation of treatment. The National Health Service in the United Kingdom recommends a maximum wait of 30 days between referral from the general practitioner to the start of treatment [9], with other authors also recommending this same limit [24].

### Treatment interruptions, compensations, and overall treatment time (OTT)

Treatment interruptions are another important indicator of quality that can have a marked effect on outcomes—especially local control—due to prolongation of radiotherapy [25, 26]. It is essential to minimize treatment prolongation whenever possible, especially when the cause of the interruption is related to easily anticipatable organizational factors. Public holidays and technical issues (unplanned quality control checks or machine malfunction) are the most common causes of interruptions [21]. Since holidays are known in advance, they should be accounted for during treatment planning. Establishing a protocol to compensate for interruptions is essential to standard practice.

In our study, treatment interruptions were common (44% overall), but highly variable among centres. The vast majority of interruptions (90.7% of cases) were due to centre-related issues. However, the differences between centres were notable: in one centre, only 2.5% of cases experienced a treatment interruption versus nearly 90% of cases at some other centres. This variability may be an anomaly (e.g., a significant machine malfunction during the study period) or it may indicate a need to implement measures to ensure that the likelihood of such interruptions is minimized. Indeed, the importance of avoiding treatment interruptions is underscored by studies showing that patients with cancer who miss ≥ two radiation therapy sessions have worse outcomes [27]. In this regard, it is worth emphasizing that none of the patients in our cohort who received SCRT experienced an interruption, a finding that supports the use of SCRT over LCRT, assuming treatment outcomes are equivalent.

Overall, compensation rates for missed fractions were low (approximately 2%). Interestingly, four centres did not compensate for any missed fractions, possibly because the interruption was limited in time (e.g., only 1 or 2 days). It is worth noting that only two of the six centres in this study work on Saturdays and can thus compensate for interruptions during the weekend. Treatment was completed in the prescribed time in just over half of cases, reflecting the high interruption rate, particularly in Spain.

### Follow-up

Follow-up and survivorship care has become a major area of interest in cancer treatment [28], not only due to the risk of recurrence and assessment of late side effects, but also because patients in the follow-up stage have a distinct range of physiological, psychosocial, and functional needs which require frequent contact with the team of healthcare professionals. According to NCCN guidelines, patients treated for stage II or III colorectal cancer should undergo CEA testing every 3 to 6 months for 2 years and then every 6 months for 3 additional years, plus CT scans every 6 to 12 months for 5 years. In the present study, follow-up was considered “regular” if the patient was seen at least twice per year. Based on this definition, follow up was satisfactory in most cases (Table 4).

### Adverse effects

The rate of ≥ grade 3 adverse effects (AE) was relatively low in this cohort (Table 5). However, given that most patients who undergo pelvic radiation will present symptoms of acute radiation-induced bowel injury [29], the relatively low rate of acute AEs probably reflects under-reporting, a finding that highlights another area to target for improvement to ensure that all AEs are graded and registered on the medical record. Nevertheless, these findings are not unusual and reflect a widespread tendency for underreporting of late toxicity, which has been attributed in part to patients’ unwillingness to report mild chronic symptoms (such as loose stools and diarrhoea) or “embarrassing” symptoms such as flatulence and faecal incontinence/leaking [29]. Clearly, there is a need for a more systematic approach to registering adverse effects, which would also provide internationally comparable data [30].

### Study strengths and limitations

This study has several limitations. First, we evaluated a maximum of 40 clinical records per centre, which may be insufficient to draw any definitive conclusions. Another limitation is missing clinical data at some centres, mainly attributable to organizational/technical issues such as limited access to patient data (one centre only had access to data reported by the radiation oncologist) or the use of paper-based medical records (one centre). In addition, due to the retrospective study design, some variables with a potential clinical impact could not be assessed, including the time from onset of symptomatology to anatomopathologic confirmation and treatment initiation. By contrast, the main strengths of this study are the multi-institutional, international design and the numerous quality indicators assessed. To our knowledge, this clinical audit contains the largest series of rectal cancer patients treated with radiotherapy to date (Table 6).

The IROCA group will use these findings presented here to develop a plan of action to ensure that all participating centres meet criteria for standard clinical practice and to harmonise the best practices identified at each institution, which will then be evaluated in a future clinical audit.

## Conclusion

The present clinical audit, performed at six comprehensive cancer care centres in Europe, shows that most centres adhered to standard clinical practice for most of the quality indicators evaluated. However, the audit also revealed substantial variability in clinical practice and multiple targets for improvement were identified, most notably the need to ensure that all relevant data are recorded on the medical records, particularly the presence and grade of all adverse effects. These findings confirm the value of performing multi-institutional audits to detect suboptimal adherence to standard clinical practice. Systematic monitoring, including the use of routine clinical audits, is essential to detect potential deviations from standard clinical practice in order to implement changes to improve clinical radiotherapy processes and, thereby, patient outcomes.

## Data Availability

The datasets used and/or analysed during the current study are available from the corresponding author on reasonable request.

## Abbreviations

IROCA: Improving Quality in Radiation Oncology through Clinical Audits
WCO: Wielkopolskie Centrum Onkologii
IPO: Instituto Português de Oncologia
UNIUPO: Università degli Studi del Piemonte Orientale
ICO: Institut Català d’Oncologia
ICO-H: ICO-Hospitalet
ICO-B: ICO-Badalona
ICO-G: ICO-Girona
MTB: Multidisciplinary tumour board
SCRT: Short-course radiotherapy
LCRT: Long-course radiotherapy
VMAT: Volumetric arc radiotherapy
IMRT: Intensity-modulated radiotherapy
SIB: Simultaneous-integrated boost
EBRT: External beam radiotherapy
AE: Adverse effects
3DRT: Three-dimensional radiotherapy
OTT: Overall treatment time
